# Age-related changes in factors associated with self-rated health in Swedish older adults– findings from the Gothenburg H70 study cohort born 1930

**DOI:** 10.1186/s12877-025-05923-8

**Published:** 2025-04-24

**Authors:** Karolina Thörnqvist, Lena Johansson, Maria Solevid, Anne Ingeborg Berg, Ingmar Skoog, Hanna Falk Erhag

**Affiliations:** 1 Neuropsychiatric Epidemiology Unit, Institute of Neuroscience and Physiology, Department of Psychiatry and Neurochemistry, Sahlgrenska Academy, University of Gothenburg, Gothenburg, Sweden; 2 Centre for Ageing and Health (AgeCap), University of Gothenburg, Gothenburg, Sweden; 3 Institute of Health and Care Sciences, Sahlgrenska Academy, University of Gothenburg, Gothenburg, Sweden; 4 Department of Political Science, University of Gothenburg, Gothenburg, Sweden; 5 Department of Psychology, University of Gothenburg, Gothenburg, Sweden; 6 Region Västra Götaland, Sahlgrenska University Hospital, Psychiatry Cognition and Old Age Psychiatry Clinic, Gothenburg, Sweden

**Keywords:** Aging, Self-rated health, Health transition, Younger-old, Older-old

## Abstract

**Background:**

Knowledge about age-related changes in factors associated with self-rated health (SRH) in older adults is still limited.

**Objective:**

To explore changes in SRH and analyze the strength of the association between different factors and SRH at ages 70, 75, 85, and 88 in a cohort born in 1930.

**Design:**

Cross-sectional.

**Setting:**

The Gothenburg H70 Birth Cohort Studies.

**Subjects:**

939 individuals, providing 1841 observations.

**Method:**

SRH was assessed using the same question at every examination. Factors potentially affecting SRH included somatic and mental disease burden, functional ability, life satisfaction, and loneliness. Lung function was included as an indicator of physical fitness. Descriptive statistics and binary regression were used to explore cohort characteristics, associated factors, and SRH. GLMM (Generalized linear mixed model) was used to perform a sensitivity analysis and test the robustness of our results.

**Results:**

There was an association between factors and SRH at every age, except feelings of loneliness and having a low disease burden at 85. High disease burden showed the strongest association at 70 and the weakest at 85. Depression showed the strongest association at 85 and the lowest at 88. When also controlling for life satisfaction, the associations changed between the ages, and feelings of loneliness were no longer associated with poor SRH other than at 88. The association between factors and poor SRH was generally stronger at ages 70–75 than at ages 85–88. The sensitivity analysis using GLMM confirmed the robustness of our results.

**Conclusion:**

The proportion of poor SRH decreases with age despite a higher frequency of somatic health conditions. Associated factors varied across ages, indicating that physical factors more strongly influence SRH in younger-old adults, while psychosocial factors have a greater impact on SRH in older-old adults.

**Supplementary Information:**

The online version contains supplementary material available at 10.1186/s12877-025-05923-8.

## Background

Self-rated health (SRH) is a global self-assessment of an individual’s health status and is considered a strong predictor of disability, morbidity, and mortality [1–3]. With the question, “In general, would you say your health is excellent, very good, good, fair, or poor?” (or some variations thereof), SRH delegates the task of synthesizing the many dimensions that make up the complex concept of health to the individual respondent. SRH allows us to capture elements that more guided questions cannot [4–6]. However, our understanding of factors influencing a person to give a poor or excellent health rating is still limited. Jylhä’s [4] well-cited conceptual model describes SRH as the result of a complex evaluation process that considers health-related factors, such as medical diagnoses, functional status, and symptom experience, as well as the contextual frameworks of evaluation, such as age, culture, comparison mechanisms, references, and personal disposition [4]. The model implies that the importance of some evaluation criteria might change with age (i.e., response shift) and that different groups use the response options differently.

Previous studies have identified chronic illness, multimorbidity [1, 7, 8], functional impairment [1, 9], psychological well-being [1, 10], physical activity [11], cognitive function [12], depressive symptoms [13, 14], positive affect [15, 16], life satisfaction [17], as well as demographic factors [4, 18–21] as significant determinants of SRH in older adults. However, as we age, studies also suggest that the link between symptoms, diagnosed conditions, and functional status, on the one hand, and SRH, on the other hand, changes [8]. There also seems to be a shift in the relative importance of factors affecting SRH, with psychosocial factors becoming more important with age, potentially mitigating the adverse effects of illness and functional decline [22].

When comparing SRH in younger-olds (i.e., 65–75) with older-olds (i.e., > 85 years), studies show that the association between subjective and objective health weakens with advancing age [22–24]. Previous research indicates that the gap between objective health and SRH continues to increase with age [25]. This relationship has been identified even in the absolute oldest age groups above age 100 [26]. This paradox is commonly explained by coping skills and decreased health aspiration levels, allowing health to be experienced as satisfactory even if it is worse than before [5, 8, 27, 28]. However, few studies have longitudinally examined individual transitions in SRH of older adults [29], and a closer look at age-related changes in SRH is needed. This study aims to explore changes in SRH and analyze the strength of the association between the different factors and SRH at ages 70, 75, 85, and 88 in a cohort born in 1930.

## Methods

### Study population

**Fig. 1:**
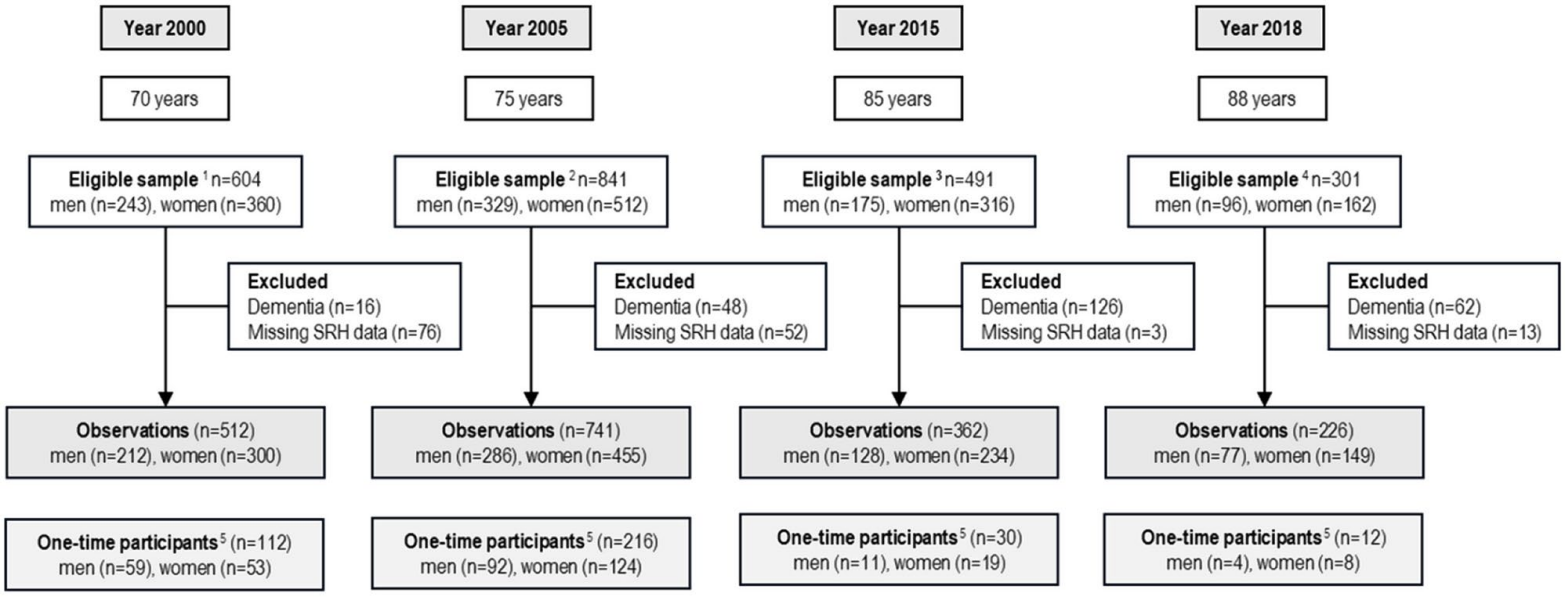
Sample flow-chart describing the four examination waves. ^1^The sample consisted of all 70-year-olds born in 1930 as well as participants previously examined in the Gothenburg Women's Study born in 1930. ^2^ The sarnpleconsisled of participants previously examined at age 70 as well as new 75-years-olds born in 1930 living in Gothenburg (n=384). ^3^ The sample consisted of participants previously examined at ages 70 and 75 as well as new 85-year-olds bom in 1930 living in Gothenburg (n=67). ^4^ The sample consisted of participants previously examined at ages 70, 75, and 85 years as well as new 88-year-olds born in 1930 living in Gothenburg (n=14).^5^ Individuals only participating at one of the examinations

This study is a part of the Gothenburg H70 Birth Cohort Studies in Sweden (the H70 studies). The complete study protocol is described elsewhere [30]. All study participants were registered residents in Gothenburg born on pre-selected birth dates. Information regarding date of birth and residential address (both in ordinary and special housing) was obtained from the Swedish Tax Agency’s population registry. In this study, we use data from the 1930 cohort where participants were examined at ages 70 (*n* = 512), 75 (*n* = 741), 85 (*n* = 362), and 88 (*n* = 226) years (Fig. 1). Due to a loss to follow-up, new participants were added to the 1930 cohort in 2005, 2015, and 2018, resulting in varying numbers of examinations per participant. Some participated in one examination, whereas others participated in all four examination waves. In total, 939 unique participants at ages 70, 75, 85, and 88 years yielded the 1841 observations used in this study (Supplemental Table 1). In the present study, participants with dementia were excluded (*n* = 252) due to the risk of reporting bias and misinterpretation. The study was approved by the Regional Ethical Review Board in Gothenburg (approval numbers: 240800/ S227-00, 041104/T453-04, 270415/131 − 15 and 230418/278 − 18).

### Self-rated health

SRH was assessed using the same question at every examination. At ages 70 and 75, the participants rated their health on a five-point scale (i.e., “excellent,” “very good,” “good,” “moderate,” and “poor”) while at ages 85 and 88, they rated their health on a four-point scale (i.e., “very good,” “good,” “poor,” and “very poor”) with higher values indicating better SRH. To harmonize the response options between examinations, we collapsed “excellent,” “very good,” and “good” into “Good SRH,” and “fair,” “poor,” and “very poor” into “Poor SRH.”

### Demographic factors

Educational level was dichotomized as equal to or less than compulsory education (i.e., six years) or more than compulsory (i.e., at least one more year). Marital status was dichotomized as married/cohabiting or living alone.

### Somatic health factors

Somatic health conditions were self-reported and ascertained by a positive answer to the question, “Have you ever been told by a doctor that you have…?” In the present study, we included cardiovascular disease (i.e., angina pectoris, myocardial infarction, and intermittent claudication), diabetes (types 1 and 2), and stroke/TIA. Urinary incontinence (yes/no) and joint pain (yes/no) were also included. Lung function was measured with Peak Expiratory Flow (PEF), and a cut-off for normal lung function in older adults was set at > 220 L/minute for women and 350 L/minute for men [31]. The number of conditions was summed into a total score and then categorized into no/minimal disease burden (0–1 somatic health problems), low disease burden (2–3 somatic health problems), and high disease burden (≥ 4 somatic health problems).

### Mental health factors

In a psychiatric interview, the participants were asked questions about their mental health, and major depression was identified according to the Diagnostic and Statistical Manual of Mental Disorders Fifth Edition [32], and minor depression according to DSM-IV-TR research criteria [33]. For this study, “any depression” was used to denote those fulfilling the requirements for either major or minor depression.

### Functional factors

Activities of daily living (ADL) included feeding, dressing, showering, going to the toilet, getting up from a chair, and indoor and outdoor mobility. Instrumental activities of daily living (IADL) included caring for one’s home. ADL and IADL were coded as either independent (including the use of aids) or dependent (in need of help from another person). The ADL and IADL activities were summed into a total score, and participants with ≥ 1 ADL/IADL dependency were grouped into “any ADL/IADL disability.”

### Psychological factors

Level of satisfaction in five life domains (living conditions, social relationships, leisure time, economy, and total life situation) was graded on a 7-point scale and summed to an index score ranging between 5 and 35 points, with higher values indicating a higher level of life satisfaction (Cronbach’s alpha > 0.7 at all four examination points). Feelings of loneliness were rated as either yes or no.

### Statistical analysis

Descriptive statistics were used to report sample characteristics and differences in proportions across examination occasions and sexes. Life satisfaction was treated as a continuous variable, and all other variables were categorical. Binary regression analysis was used to test the association between the different factors and SRH. In the first model, the associations were tested separately at each time point, controlling for sex and educational level. In the second model, we also adjusted for life satisfaction. Two-tailed p-values of < 0.05 were considered statistically significant. To test the robustness of the binary regression analysis, we conducted a sensitivity analysis using a Generalized Linear Mixed Model (GLMM). As in the binary regression analysis, we ran two models: the first controlled for sex and educational level, while in the second, life satisfaction was also included to adjust the analysis. The first analysis was performed using IBM SPSS Statistics 29.0.00. The sensitivity analysis was performed using RStudio.

**Table 1:** Characteristics of the sample at each examination (*n*=1841)

	Age 70 *n* = 512	Age 75 *n* = 741	Age 85 *n* = 362	Age 88 *n* = 226	*P*-value for trend
Women, *n* (%)	300 (58.6)	455 (61.4)	234 (64.6)	149 (65.9)	0.156
Educational level, more than compulsory ^a)^, *n* (%)	207 (40.5)	349 (47.1)	189 (53.4)	121 (53.8)	< 0.001
Living alone ^b)^, *n* (%)	178 (34.8)	311 (42.0)	236 (65.4)	160 (72.7)	< 0.001
Poor SRH	95 (18.6)	225 (30.4)	59 (16.3)	34 (15.0)	< 0.001
Somatic health factors;					
No/minimal somatic disease burden (0–1 health problems), n (%)	271 (52.9)	340 (45.9)	122 (33.7)	78 (34.5)	< 0.001
Low somatic diseased burden (2–3 health problems) *n* (%)	212 (41.4)	330 (44.5)	164 (45.3)	118 (52.2)	< 0.001
High somatic disease burden (≥ 4 health problems), *n* (%)	29 (5.7)	71 (9.6)	76 (21.0)	30 (13.3)	< 0.001
Any depression ^c)^, *n* (%)	62 (12.1)	148 (20.2)	46 (12.8)	39 (17.4)	< 0.001
Any ADL/IADL dependency ^d)^, *n* (%)	46 (9.0)	98 (13.3)	116 (32.0)	117 (51.8)	< 0.001
Life satisfaction ^e)^, mean (SD)	30.19 ± 4.8	29.02 ± 5.3	29.54 ± 5.1	29.19 ± 5.7	0.001
Feeling lonely, *n* (%)	89 (17.8)	128 (17.6)	92 (25.6)	89 (40.3)	< 0.001

Educational level was dichotomized as equal or less than compulsory education (i.e., six years) or more than compulsory (i.e., at least one more year)

Marital status was dichotomized as married/cohabiting or living alone

“Any depression” was used to denote those fulfilling the criteria for either major or minor depression

ADL/IADL included feeding, dressing, showering, going to the toilet, getting up from a chair, indoor and outdoor mobility, and caring for one’s home

Level of satisfaction regarding living conditions, social relationships, leisure time, economy, and total life situation was graded on a 7-point scale and summed to a total score ranging between 5 and 35 points, with higher values indicating a higher level of life satisfaction

Pearson Chi-Square was used to test categorical group differences, and ANOVA was used to test the variance in life satisfaction. Data source– https://www.gu.se/forskning/epinep

## Results

There were 939 individuals in the sample that provided 1841 observations at ages 70, 75, 85, and 88. Participants were examined on either one (*n* = 370), two (*n* = 326), three (*n* = 153), or four (*n* = 90) occasions (Supplemental Table 1). The characteristics of the participants at each examination are presented in Table 1. There was a difference between examinations regarding all characteristics, except in the proportion of women. The proportion of participants living alone, feeling lonely, having a high disease burden (≥ 4 health problems), and being dependent in ADL/IADL increased with age.

The proportion of participants rating their health as poor differed between the ages, with the highest proportion observed at age 75 (30%) and the lowest at age 88 (15%) (Table 1). At age 75, women more often reported poor SRH (men 25% vs. women 34%, *p* = 0.015). The original response options used at ages 70 and 75 and at ages 85 and 88, respectively, are presented in Supplemental Table 2. Although statistically significant, the difference between ages in life satisfaction was minimal.

The associations between the different factors and poor SRH, adjusted for sex and educational level, are presented in Table 2. All included factors showed a statistically significant association with poor SRH at every age, except low somatic disease burden at ages 85 and 88 and feeling of loneliness at age 85. At ages 70, 75, and 88, the strongest association with poor SRH was found for high somatic disease burden. At age 85, depression showed the strongest association with poor SRH. ADL/IADL dependency had the second strongest association with poor SRH across all ages.

When comparing the strength of the association between ages, both high and low somatic disease burden showed the strongest association with poor SRH at age 70, high somatic disease burden was weakest at age 85, and low somatic disease burden wasn’t significant at either age 85 or 88. ADL/IADL dependency showed the strongest association with poor SRH at age 70 and the weakest at age 85. Depression showed the strongest association with poor SRH at age 85 and the weakest at age 88. Feeling lonely showed the strongest association with poor SRH at age 88 and the weakest at age 75. Higher life satisfaction decreased the probability of poor SRH at every age, with the highest probability observed at age 70 and the lowest at age 88.

**Table 2:** The association between factors and poor SRH at different ages, controlling for sex and educational level

	Age 70 *n* = 512	Age 75 *n* = 741	Age 85 *n* = 362	Age 88 *n* = 226
	OR (95% CI)	OR (95% CI)	OR (95% CI)	OR (95% CI)
Low somatic disease burden (2–3 health problems)	3.35 (1.97–5.68)*	3.13 (2.16–4.53)*	1.17 (0.57–2.41)	2.74 (0.97–7.76)
High somatic disease burden (≥ 4 health problems)	25.31 (10.18–62.93)*	11.39 (6.32–20.49)*	2.75 (1.28–5.91)*	7.74 (2.32–25.80)*
ADL/IADL dependency	5.78 (3.04–10.99)*	5.63 (3.55–8.92)*	4.22 (2.30–7.75)*	4.67 (1.92–11.40)*
Depression	4.79 (2.68–8.56)*	4.24 (2.89–6.22)*	5.33 (2.65–10.74)*	3.18 (1.39–7.27)*
Life satisfaction	0.79 (0.75-0.84)*	0.85 (0.82-0.88)*	0.83 (0.77-0.89)*	0.87 (0.81-0.93)*
Feeling lonely	2.53 (1.48–4.33)*	1.84 (1.24–2.76)*	1.79 (0.95–3.35)	3.03 (1.39–6.61)*

Binary Logistic Regression was used to test the association between SRH and related factors at different ages. Data source– https://www.gu.se/forskning/epinep

* Significant associations, P-value < 0.05

In the first model, life satisfaction reduced the probability of poor SRH at all ages. Life satisfaction was then entered as a control factor in the second model to test its protective effect on SRH. When also controlling for life satisfaction, the associations between factors and poor SRH changed between the ages, and feelings of loneliness were no longer associated with poor SRH other than at age 88 (Table 3). The association between depression and poor SRH remained strongest at age 85 despite the association decreasing at all ages. The association between low somatic disease burden and poor SHR decreased at ages 70 and 75 and between high disease burden and poor SRH at ages 70 and 75. However, the association between high disease burden and poor SRH increased at age 85, while the association with ADL/IADL dependency decreased at all ages. The associations between the different factors and poor SRH, adjusted for sex, educational level, and life satisfaction, are presented in Table 3.

**Table 3:** The association between factors and poor SRH at different ages, controlling for sex, educational level, and life satisfaction

	Age 70 *n* = 512	Age 75 *n* = 741	Age 85 *n* = 362	Age 88 *n* = 226
	OR (95% CI)	OR (95% CI)	OR (95% CI)	OR (95% CI)
Low somatic diseased burden (2–3 health problems)	2.92 (1.62–5.26)*	2.61 (1.75–3.90)*	1.93 (0.67-5.56)	1.90 (0.57-6.41)
High somatic disease burden (≥ 4 health problems)	14.62 (5.33–40.06)*	8.45 (4.45–16.01)*	4.40 (1.50-12.91)*	5.51 (1.45–20.91)*
ADL/IADL dependency	4.74 (2.23–10.05)*	4.04 (2.42–6.73)*	2.73 (1.28–5.84)*	2.87 (1.10–7.48)*
Depression	2.01 (1.01–3.99)*	2.38 (1.54–3.67)*	3.72 (1.54–8.98)*	2.65 (1.00-6.99)*
Feeling lonely	1.11 (0.58 − 2.10)	0.93 (0.58 − 1.50)	1.19 (0.52-2.72)	2.48 (1.03–5.99)*

Binary Logistic Regression was used to test the association between SRH and related factors at different ages. Data source– https://www.gu.se/forskning/epinep

* Significant associations, P-value < 0.05

### Sensitivity analysis

To assess the robustness of our results, we conducted a sensitivity analysis using a generalized linear mixed model (GLMM). This approach allowed us to account for potential within-subject correlations by including participant ID as a random intercept, thereby controlling for intra-individual variability across repeated observations.

As in the binary regressions, we ran two models: the first model adjusted for sex and educational level, and the second model was also adjusted for life satisfaction. The results from the GLMM analysis, presented in Supplement Table 3, were generally consistent with the initial logistic regression findings. However, the inclusion of interaction terms in the GLMM provided additional insight into age-related variations. Compared to the reference group at age 70, the associations between SRH and health-related factors generally strengthened at age 75, suggesting a peak in these relationships. However, by the ages of 85 and 88, the associations weakened or were no longer statistically significant, indicating a possible age-related decline in their relevance. In line with our initial analysis, the association between poor SRH and high somatic disease burden was reduced at ages 85 and 88, compared to younger age groups.

In the binary regression, loneliness showed a stronger association with poor SRH at age 88 compared to age 70. However, in the GLMM, the overall association between loneliness and SRH was not significant, and no clear age-related interaction was observed. The GLMM analysis, which included adjustments for sex, educational level, and life satisfaction (Supplement Table 4), confirmed the overall trend observed in the binary regression: associations between SRH and health-related factors generally weakened when life satisfaction was added in the analysis.

## Discussion

In this study, we aimed to explore changes in SRH and analyze the strength of the association between the different factors and SRH at ages 70, 75, 85, and 88 in 939 individuals, providing 1841 observations. The results showed that somatic disease burden, being ADL/IADL dependent, living alone, and feeling lonely increased with age, although the proportion with depression was largest at age 75. Only 15% reported poor SRH in the oldest age group (88 years), but as many as 30% at age 75. Although SRH, somatic, and mental health fluctuated over time, the level of life satisfaction remained unchanged.

While the participants rated their SRH better at age 88 than 75, there was an age-related increase in several factors used in this study. It is possible that resilience, successful coping, and acceptance of one’s current health and functional status made the surviving participants more health-positive with increasing age [29, 34]. Resilience is crucial in how individuals adapt to and cope with chronic health conditions, enabling them to manage challenges more effectively [35]. Higher levels of resilience might help mitigate the risk of functional disabilities and buffer the impact of new health issues [36]. Previous studies have shown that SRH remains stable over time in older ages [37, 38]. Despite higher levels of disease burden, older-olds tend to rate their health as good, perhaps due to successful adaptation strategies enabling them to cope with their illnesses and functional disability [8, 39, 40]. In our study, only 15% of the 88-year-olds rated their health as poor compared to 30% of the 75-year-olds.

Consistent with previous studies [22, 23], our results showed that controlling for sex and educational level, the association between factors and SRH shifted between the ages. At ages 70, 75, and 88, high somatic disease burden showed the strongest association with poor SRH. In contrast, at age 85, depression showed the strongest association with poor SRH. Although our results indicate a shift between somatic and mental factors, being dependent on others in everyday life showed the second strongest association with poor SRH at all ages, especially at ages 85 and 88. Autonomy and the ability to function independently are essential to a person’s sense of control, self-efficacy, and self-determination, and previous studies have shown a relationship between being able to manage independently in everyday life and a sense of well-being in older adults [41, 42].

Our results showed that a high level of life satisfaction was associated with better SRH at all ages, although the proportion of participants with a high disease burden and functional disability increased with age. In contrast to previous studies showing that the association between life satisfaction and SRH increases with age [22], our results indicated that life satisfaction remained essentially unchanged between ages. When controlling for life satisfaction, poor SRH was no longer associated with feelings of loneliness, other than at age 88, and the association with depression declined at all ages. Also, the association between somatic disease burden and ADL/IADL dependency decreased. Both SRH and life satisfaction reflect the individual’s reflective judgment, and a possible explanation as to why controlling for life satisfaction affected the associations between factors and SRH might be that life satisfaction and SRH overlap. Previous studies have shown a mutual mediating relationship between SRH and life satisfaction, where good SRH indicates a higher life satisfaction score and vice versa [43, 44].

Quite notably, when controlling for life satisfaction, the association between functional disability and poor SRH at age 88 disappeared. Although there might be several explanations for this finding, it indicates that psychosocial factors become more important with age. Older adults who experience high levels of contentment in areas indirectly related to health, such as social relationships (e.g., marital status, children or friends), living conditions, lifestyle, and income, are possibly better equipped to cope with both current health conditions and upcoming health problems [45, 46].

The sensitivity analysis, conducted using the Generalized Linear Mixed Model (GLMM), confirmed the robustness of our findings. While individual and age-related variability may influence specific estimates, the overall conclusions remained consistent. Compared to the binary regression, the GLMM provides a more nuanced understanding by accounting for intra-individual variability. This allowed us to identify a general decline in the strength of associations between health-related factors and self-rated health (SRH) with advancing age. Poor SRH remained strongly associated with somatic disease burden, functional ability, and depression across models. The observed age-related pattern is consistent with the binary regression results, which also showed stronger associations at age 75 and weaker or non-significant associations at ages 85 and 88. However, the GLMM more clearly illustrates how intra-individual variability contributes to this attenuation across age groups.

Taken together, these findings support the validity of SRH as a broad indicator of age-related health, even though its associations with some health-related factors appear to weaken in the oldest age groups.

This study has both strengths and limitations. The strengths of this study include the four-time points of cross-sectional health examinations over an 18-year follow-up period in a population-based sample of older. There are also several limitations. Firstly, due to study attrition, new participants were added to the sample at ages 75, 85, and 88, affecting the possibility of analyzing the data longitudinally. Secondly, the data used in this study is mainly cross-sectional, only allowing us to explore inter-individual changes in the association between related factors and SRH. Thirdly, the risk of selection bias increases by primarily using cross-sectional data. For instance, at age 75, new participants were included, which could lead to skewed data. Another potential risk for selection bias lies in the likelihood that participants with poor SRH either die or decline participation due to their health status, leading to a healthier sample as age increases. Fourthly, the total number of somatic health conditions and ADL/IADL dependencies were positively skewed and, therefore, categorized. If the total scores had been normally distributed, it is possible that more detailed information about the variation of values and their association with SRH could have been captured. However, it is also possible that the categorization enabled us to detect relationships that might otherwise have been missed. Fifthly, although we controlled for sex, education, and life satisfaction, group differences might affect the strength of the association between related factors and SRH. For instance, Spuling et al. [22] suggest that the association between SRH and various factors is affected not only by age but also by birth year, pointing out that different birth cohorts rate their health differently. Since we only included one birth cohort, we cannot analyze the effect of birth cohorts in this study. Sixthly, the proportion of those who rated their health as poor was two times larger at age 75 compared to age 88. A possible explanation for this difference is survival. Although the somatic disease burden and ADL/IADL dependency increased with age, there is a possibility that those examined at age 75 had a higher mortality rate than those examined at age 88, meaning that those still alive and in sufficient health to the participants in the study at age 88 was those in better health age 75. Seventhly, this study explored the association between mainly self-rated associated factors and SRH. Participants with dementia were excluded from this study due to the potential limitations of self-reported data in this population. As the disease progresses, individuals may experience significant difficulties in recalling past events or understanding the context of questions posed to them.

Lastly, although the SRH question was phrased identically between examinations, the response options varied over time. To compare SRH between ages, we collapsed the response options into two categories, potentially limiting the detection of more nuanced changes in SRH between examinations.

## Conclusion

SRH is a frequently used health measurement that evaluates individuals’ subjective health. The knowledge about SRH in later life and factors associated with individuals’ health assessments is limited, which motivates our research. The results show that the proportion of poor SRH decreases with age, despite an increase in somatic health disorders. We also identify that the associated factors varied in significance across ages, suggesting that physical factors have a more prominent role in SRH among younger-olds, while psychosocial factors are more important among older-olds. When accounting for intra-individual variability, the results indicate that the association between health-related factors and poor SRH becomes weaker with age. However, additional longitudinal research is needed to gain a deeper understanding of the changes in related factors that influence SRH changes with increasing age, and thereby identify changes over the lifespan.

## Electronic supplementary material

Below is the link to the electronic supplementary material.

Supplementary Material 1

## Data Availability

The data used in this article can be made available by the corresponding author upon request.

## Abbreviations

ADL: Activities of daily living
IADL: Instrumental activities of daily living (IADL)
GLMM: Generalized Linear Mixed Model
SRH: Self-rated health
The H70 studies: The Gothenburg H70 Birth Cohort Studies in Sweden
