# Supplementary material for: Age-related changes in factors associated with self-rated health in Swedish older adults– findings from the Gothenburg H70 study cohort born 1930
Source: BMC Geriatr. 2025 Apr 24;25:275. doi: 10.1186/s12877-025-05923-8 (PMC12023556; doi:10.1186/s12877-025-05923-8)
Supplement: Supplementary file 1 — Supplementary Material 1 [file 12877_2025_5923_MOESM1_ESM.docx]

| Supplement Table 1. Distribution of examinations sorted by age | | | | | |
| --- | --- | --- | --- | --- | --- |
| Number of examinations | Age 70 | Age 75 | Age 85 | Age 88 | Total observations |
| 1 | 112 | 216 | 30 | 12 | 370 |
| 2 | 235 | 289 | 91 | 37 | 652 |
| 3 | 75 | 146 | 151 | 87 | 459 |
| 4 | 90 | 90 | 90 | 90 | 360 |
| Total participants at each time point/age/examination (n=939) | 512 | 741 | 362 | 226 | 1841 |
| Supplement 1. Four examinations were carried out, 2000, 2005, 2015 and 2018, when the participant was 70, 75, 85 and 88 years old. This table shows the distribution of how many examinations the participants have taken part in divided by ages. | | | | | |

**Supplement Table 1.**

| Supplement Table 2. The distribution of SRH divided by sex at different ages. | | | | | | | | | |
| --- | --- | --- | --- | --- | --- | --- | --- | --- | --- |
|  | **Age 70** | | **Age 75** | |  | **Age 85** | | **Age 88** | |
| n (%)  SRH | Men  (n=212) | Women  (n=300) | Men  (n=286) | Women  (n=455) | n (%) | Men  (n=128) | Women  (n=234) | Men  (n=77) | Women  (n=149) |
| Excellent | 30 (14.2) | 25 (8.4) | 18 (6.3) | 26 (5.7) | Very good | 33 (25.8) | 48 (20.5) | 13 (16.9) | 13 (8.7) |
| Very good | 74 (34.9) | 82 (27.4) | 64 (22.4) | 84 (18.5) | Good | 79 (61.7) | 143 (64.4) | 53 (68.8) | 113 (75.8) |
| Good | 69 (32.5) | 137 (45.8) | 132 (46.2) | 192 (42.2) | Poor | 14 (10.9) | 36 (15.4) | 10 (13) | 22 (14.8) |
| Fair | 34 (16) | 52 (17.4) | 67 (23.4) | 139 (30.5) | Very poor | 2 (1.6) | 7 (3) | 1 (1.3) | 1 (.7) |
| Poor | 5 (2.4) | 3 (1) | 5 (1.7) | 14 (3.1) |  |  |  |  |  |
|  | Data source – https://www.gu.se/forskning/epinep | | | | | | | | |

**Supplement Table 2.**

| Supplement Table 3. GLMM and GLM when controlling for sex and education | | |
| --- | --- | --- |
|  | **GLMM**  **Estimate** | **GLM  Estimate** |
| **Somatic disease burden** |  |  |
| Intercept | -3.29* | -2.56* |
| Low somatic disease burden  (2-3 health problems) | 1.42* | 1.19* |
| High somatic disease burden  (≥4 health problems) | 3.75* | 3.17* |
| Agegroup75 | 0.83* | 0.65* |
| Agegroup85 | 0.42 | 0.21 |
| Agegroup88 | -0.03 | -0.35 |
| Low somatic disease burden x 75 | 0.03 | -0.01 |
| High somatic disease burden x 75 | -0.68 | -0.73 |
| Low somatic disease burden x 85 | -1.13* | -0.89 |
| High somatic disease burden x 85 | -2.62* | -2.03* |
| Low somatic disease burden x 88 | -0.36 | -0.16 |
| High somatic disease burden x 88 | -1.87* | -1.20 |
| **adl/iadl dependency** |  |  |
| Intercept | -2.66* | -1.90* |
| ADL/IADL | 2.35* | 1.82* |
| Agegroup75 | 0.85* | 0.62* |
| Agegroup85 | -0.35 | -0.55* |
| Agegroup88 | -0.67 | -0.94* |
| Adl x 75 | 0.10 | -0.64 |
| Adl x 85 | -1.02 | -0.43 |
| Adl x 88 | -0.85 | -0.35 |
| **Depression** |  |  |
| Intercept | -2.69* | -1.89* |
| Depression | 2.06* | 1.54* |
| Agegroup75 | 0.85* | 0.59* |
| Agegroup85 | -0.08 | -0.24 |
| Agegroup88 | -0.07 | -0.23 |
| Depression x 75 | -0.07 | -0.08 |
| Depression x 85 | -0.02 | 0.17 |
| Depression x 88 | -0.67 | -0.36 |
| **Life satisfaction** |  |  |
| Intercept | 6.30* | 5.15* |
| Life satisfaction | -0.30* | -0.23* |
| Agegroup75 | -1.53 | -1.70 |
| Agegroup85 | -2.79 | -1.99 |
| Agegroup88 | -4.68* | 3.75* |
| Life satisfaction x 75 | 0.08 | 0.08* |
| Life satisfaction x 85 | 0.07 | 0.04 |
| Life satisfaction x 88 | 0.15* | 0.11* |
| **Feeling lonely** |  |  |
| Intercept | -2.70* | -1.82* |
| Feeling lonely | 1.12* | 0.84* |
| Agegroup75 | 1.05* | 0.72* |
| Agegroup85 | 0.01 | -0.20 |
| Agegroup88 | -0.08 | -0.54 |
| Feeling lonely x 75 | -0.20 | -0.19 |
| Feeling lonely x 85 | -0.40 | -0.18 |
| Feeling lonely x 88 | -0.29 | -0.17 |
| Generalized Linear Mixed Model **a**djusted for sex and educational level.  Significant * = p-value <0.05 | | |

| Supplement Table 4. GLMM and GLM when controlling for sex, education and life satisfaction | | |
| --- | --- | --- |
|  | **GLMM**  **Estimate** | **GLM  Estimate** |
| **Somatic disease burden** |  |  |
| Intercept | 2.55* | 2.10* |
| Low somatic disease burden  (2-3 health problems) | 1.30* | 1.07* |
| High somatic disease burden  (≥4 health problems) | 3.05* | 2.61* |
| Agegroup75 | 0.69* | 0.54 |
| Agegroup85 | -0.58 | -0.65 |
| Agegroup88 | -0.31 | -0.50 |
| Low somatic disease burden x 75 | -0.08 | -0.09 |
| High somatic disease burden x 75 | -0.42 | -0.47 |
| Low somatic disease burden x 85 | -0.47 | -0.31 |
| High somatic disease burden x 85 | -1.50 | -1.04 |
| Low somatic disease burden x 88 | -0.63 | -0.43 |
| High somatic disease burden x 88 | -1.54 | -0.99 |
| **adl/iadl dependency** |  |  |
| Intercept | 3.54* | 2.95* |
| ADL/IADL | 2.01* | 1.58* |
| Agegroup75 | 0.68* | 0.49* |
| Agegroup85 | -0.75* | -0.81* |
| Agegroup88 | -0.75 | -0.96* |
| Adl x 75 | -0.15 | -0.17 |
| Adl x 85 | -1.11 | -0.55 |
| Adl x 88 | -1.23 | -0.66 |
| **Depression** |  |  |
| Intercept | 3.51* | 2.83* |
| Depression | 1.19* | 0.84* |
| Agegroup75 | 0.68* | 0.44* |
| Agegroup85 | -0.74* | -0.72* |
| Agegroup88 | -0.53 | -0.61* |
| Depression x 75 | -0.07 | 0.01 |
| Depression x 85 | -0.55 | 0.54 |
| Depression x 88 | -0.09 | 0.13 |
| **Feeling lonely** |  |  |
| Intercept | 4.27* | 3.45* |
| Feeling lonely | 0.31 | 0.17* |
| Agegroup75 | 0.79 | 0.54* |
| Agegroup85 | -0.68 | -0.67* |
| Agegroup88 | -0.83 | -1.05 |
| Feeling lonely x 75 | -0.37 | -0. |
| Feeling lonely x 85 | 0.06 | 0.14 |
| Feeling lonely x 88 | 0.44 | 0.77 |
| Generalized Linear Mixed Model **a**djusted for sex, educational level and life satisfaction.  Significant * = p-value <0.05 | | |
